# A case report on severe atypical bullous erysipelas induced by *Escherichia coli* in an immunosuppressed individual

**DOI:** 10.3389/fimmu.2025.1691694

**Published:** 2025-11-05

**Authors:** Xuemei Zhang, Rui Yuan, Jun Chen

**Affiliations:** ^1^ Intensive Care Unit, Hospital of Chengdu University of Traditional Chinese Medicine, Chengdu, China; ^2^ Clinical Medical College, Chengdu University of Traditional Chinese Medicine, Chengdu, China; ^3^ Department of Critical Care Medicine, Hospital of Chengdu University of Traditional Chinese Medicine, Chengdu, China

**Keywords:** immunosuppression, erysipelas bullosa, Escherichia coli, case report, severe

## Abstract

A 60-year-old female patient with a prolonged history of immunosuppression due to a 20-year condition of rheumatoid arthritis, managed with long-term glucocorticoids and immunosuppressants, developed atypical erysipelas caused by *Escherichia coli*, complicated by septic shock and multiple organ dysfunction. Clinically, she presented with abrupt onset of redness, swelling, warmth, and pain in the left lower limb, which rapidly evolved into multiple vesicles and blood-filled blisters (2–3 cm in diameter) with rupture and exudation within 24 hours, subsequently progressing to septic shock and multiple organ dysfunction. Both vesicle fluid culture and next-generation sequencing (NGS) of blood samples confirmed the presence of *Escherichia coli*. Following the initial ineffective treatment with cefuroxime, the regimen was escalated to meropenem in combination with teicoplanin. Upon confirmation of the pathogenic microorganism, the treatment was de-escalated to piperacillin/tazobactam within 24 hours, supplemented by comprehensive wound management (iodophor wet dressing, lithospermum oil application, and red light therapy) and organ support therapy. After 10 days of intensive treatment, the patient recovered, and the wound healed completely after 4 months of care. This case underscores three critical warnings: 1) the pathogen spectrum of erysipelas in immunosuppressed hosts shows a significant shift, necessitating vigilance against the possibility of Gram-negative bacterial infections, such as Escherichia coli; 2) atypical bullous skin lesions can serve as an early indicator of severe infection, with a rapid clinical course progressing to shock within 24 hours; 3) a tiered anti-infective strategy is paramount – initially broad-spectrum coverage for both Gram-negative and Gram-positive bacteria (e.g., anti-pseudomonal β-lactams plus glycopeptides), followed by de-escalation within 24 hours of pathogen identification. It is advisable to conduct early combined microbial culture and NGS testing for immunosuppressed patients presenting with skin infections, and to implement individualized broad-spectrum antibiotic regimens to enhance prognosis.

## 1 Introduction

In immunosuppressed patients, infection stands as the most prevalent complication. When their skin barrier is breached, it can give rise to severe skin and soft tissue infections, such as erysipelas or cellulitis. Such infections pose a heightened risk to patients with impaired immune function, as they are prone to progressing into necrotizing skin and soft tissue infections (NSTI), potentially leading to chronic non-healing wounds, amputation, and even death (1). The hospitalization mortality rate for these infections ranges from 9.3% to 29.3%. Should the infection advance to septic shock or multiple organ failure, the fatality rate would surge markedly (2). The typical pathogens responsible for skin and soft tissue infections are predominantly Gram-positive cocci, including streptococci and staphylococci. Nevertheless, patients with immune dysfunction may contract infections caused by Gram-negative bacilli or other uncommon pathogens, presenting challenges for early diagnosis and empirical treatment. Furthermore, immunosuppression may induce atypical skin manifestations due to immune-related toxicity, such as rare bullous lesions (e.g., atypical bullous erysipelas) (3). This type of lesion deviates from the standard bullous diseases that arise from autoimmune or genetic mechanisms, posing significant diagnostic challenges and a high risk of misdiagnosis. If the empirical antibiotics administered do not encompass the offending pathogen, it may signal a grim prognosis, underscoring the urgent need for early and precise diagnosis and treatment to forestall severe complications. This article presents a severe case study of atypical erysipelas, caused by Escherichia coli, in a rheumatoid arthritis patient with a history of prolonged steroid use, which was further complicated by multiple organ dysfunction. The objective of this report is to enhance the understanding of the pathogen spectrum of erysipelas in immunosuppressed patients and offer clinical insights for early empirical treatment.

## 2 Case description

### 2.1 Medical records

The patient, a 60-year-old female, presented with symptoms of redness, swelling, heat, and pain in her left lower limb for one day and worsening accompanied by fever for four hours, prompting her admission to the General Medicine Department at 19:03 on November 30, 2024, with a preliminary diagnosis of “lower limb skin infection” by the emergency department. Subsequently, at 02:00 on December 1, due to the progression of her condition to “septic shock,” she was transferred to the Intensive Care Unit (ICU). One day prior to admission, the patient experienced these symptoms in her left lower limb without any apparent precipitating factors. Four hours before presentation, her symptoms intensified, with a temperature rise to 38.6°C. Multiple blisters and blood blisters, measuring 2–3 cm in diameter, emerged on her left lower limb, some of which ruptured, discharging a light yellow exudate and exhibiting subcutaneous hemorrhage, resulting in severe limb swelling and an inability to walk. An ultrasound examination of both lower limbs’ arteriovenous system conducted by the emergency department revealed no significant abnormalities. The patient has a 20-year history of “rheumatoid arthritis” and has been chronically medicated with “methylprednisolone 10mg po qd, leflunomide 20mg po qd, hydroxychloroquine 0.2g po bid, and alfacalcidol 0.25mg po qd”.

**Figure 1:**
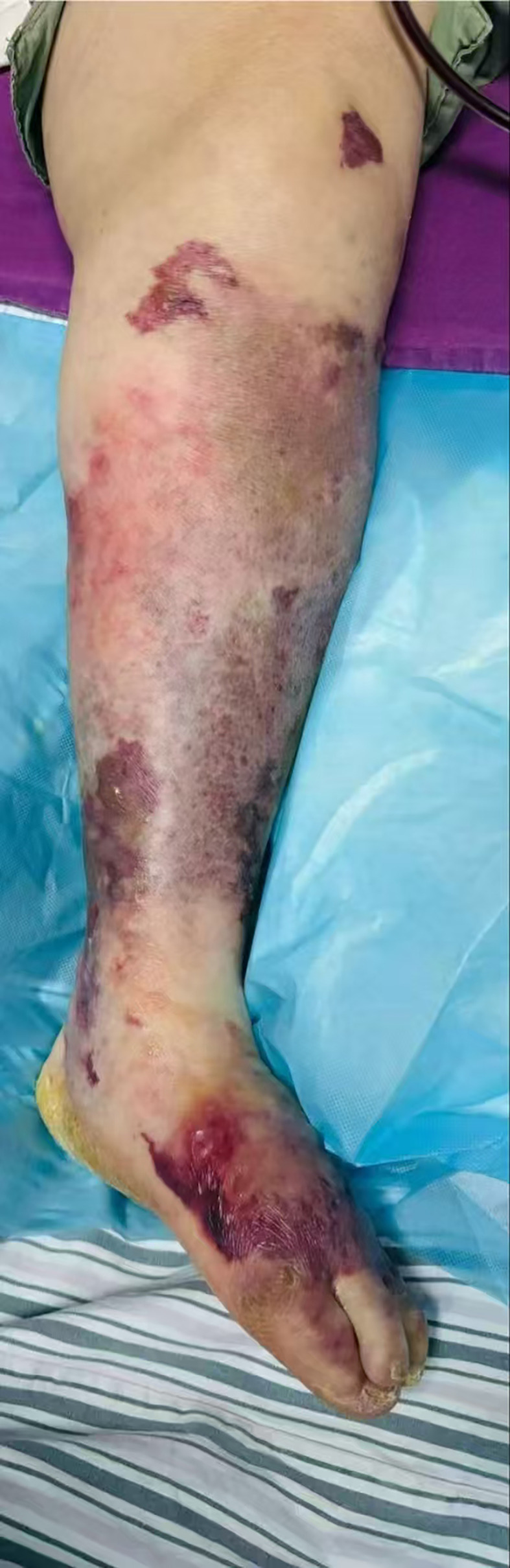
Frontal view of left lower limb. Skin manifestations: diffuse erythema and swelling, elevated skin temperature, along with multiple irregular blisters and blood blisters (1–2 cm in diameter) accompanied by visible subcutaneous ecchymosis.

**Figure 2:**
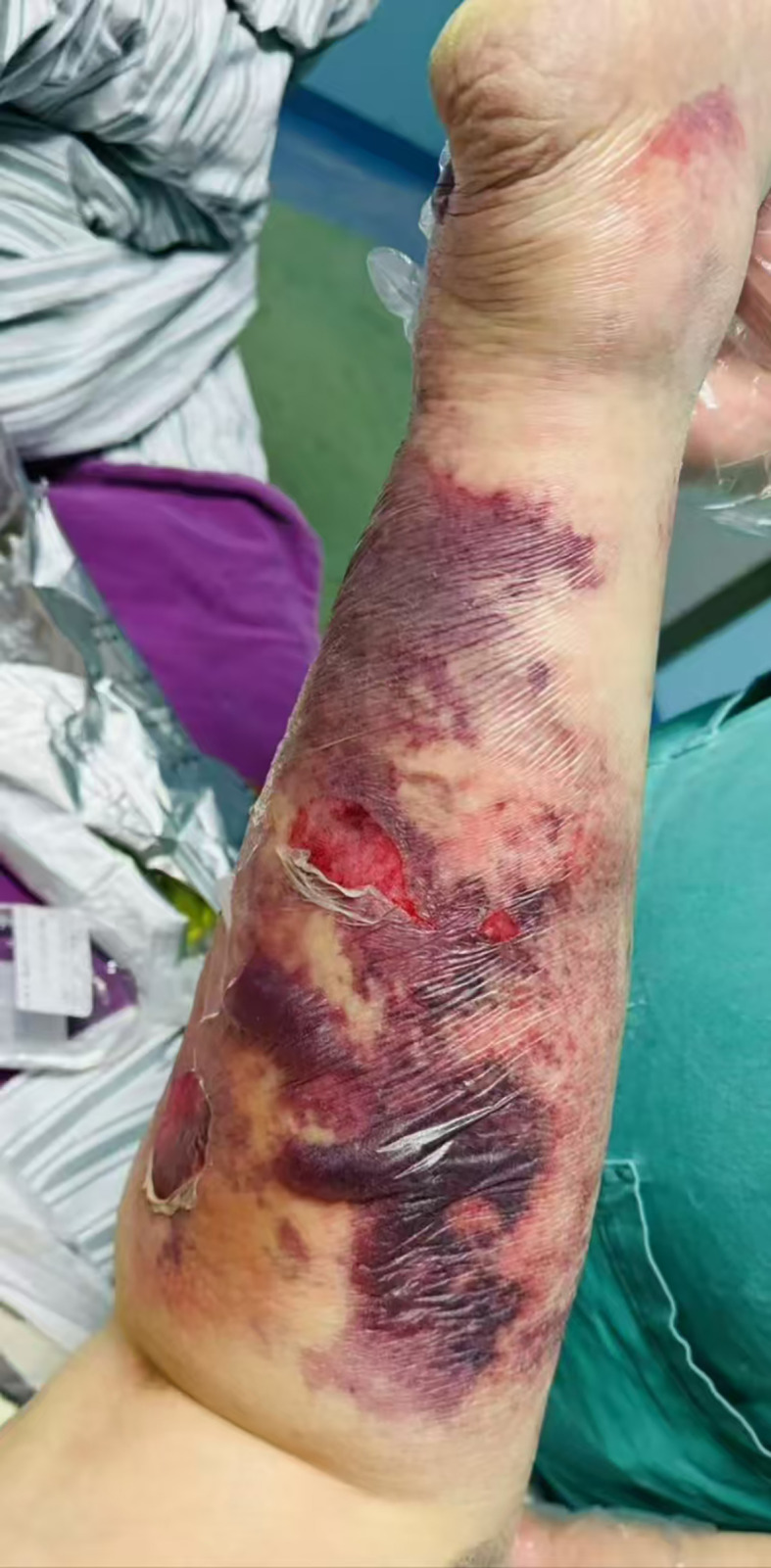
Dorsal view of left lower limb. Skin manifestations: The skin appears thin, with diffuse redness and swelling, elevated skin temperature, and multiple irregular blisters and blood blisters (2–3 cm in diameter) that are ruptured, oozing fluid and blood, and accompanied by subcutaneous ecchymosis.

Physical examination upon admission revealed: Temperature at 36.6°C, pulse rate at 128 beats per minute, respiratory rate at 25 breaths per minute, and blood pressure at 128/64 mmHg. The patient was conscious but agitated, with an obese body shape, a moon face, a buffalo hump, edema on the face and both lower limbs, and delicate skin all over. There was diffuse redness, swelling, heat, and pain extending from the calf to the dorsum of the foot on the left lower limb, accompanied by multiple irregular vesicles and blood blisters (2–3 cm in diameter) with ulcerations, exudates, bleeding, and subcutaneous ecchymoses (as shown in **Figures 1**, **2**).

The relevant auxiliary examinations after admission are shown in **Table 1**.

**Table 1:** Key laboratory and imaging examination results.

Inspection items		Result	Unit	Reference range
Inflammatory indicators	C-reactive protein (CRP)	334	mg/L	0-10
	Procalcitonin (PCT)	>50	ng/mL	<0.05
	Interleukin-6 (IL-6)	>5500	pg/mL	<7
	White blood cell count (WBC)	8.4	10^9^/L	4-10
Organ function	Creatinine (Crea)	189.5	μmol/L	44-133
	Estimated glomerular filtration rate (eGFR)	24.3	ml/min/1.73m^2^	>90
	Myoglobin (MYO)	>1000	ng/mL	<110
	N-terminal pro-B-type natriuretic peptide (NT-pro-BNP)	11823.2	pg/mL	<125
	Aspartate aminotransferase (ALT)	58	U/L	1-40
Blood Gas Analysis	Arterial blood lactate (Lac)	6.3	mmol/L	0.5-1.6
	Arterial blood partial pressure of carbon dioxide (PCO_2_)	20	mmHg	35-45
	Arterial partial pressure of oxygen (PO_2_)	210	mmHg	80-100
	Bicarbonate ion (HCO_3_^-)^	14.2	mmol/L	21.4-27.3
	Remaining Alkali (BE)	-9.6	mmol/L	-3-3
Etiology	Bacterial culture of vesicle fluid	*Escherichia coli* (ESBL-negative) Resistant to moxifloxacin, ciprofloxacin, levofloxacin, ticarcillin, sulfamethoxazole, tetracycline, sensitive to cefepime, ceftazidime, ceftizoxime, resistant to piperacillin, sensitive to piperacillin/tazobactam, sensitive to cefoperazone/sulbactam, sensitive to meropenem, imipenem, sensitive to amikacin.	/	sterile
	Blood t-NGS	*Escherichia coli* Sequence number 65	/	feminine
Imaging	Chest and abdomen CT	Chronic bronchitis with signs of emphysema; scattered patchy shadows and streaky foci in both lungs, infection?	/	/
Rheumatic immunity	rheumatoid factor	21.9	IU/mL	<20
	Immunoglobulin G (IgG)	3.76	g/L	7-16
	Immunoglobulin M (IgM)	0.17	g/L	0.4-2.3
	Complement C3	0.68	g/L	0.9-1.8
Hormonal indicators	Cortisol (COR)	>600	ng/ml	57.2-194.2
	Thyroxine (T4)	58	nmol/L	66-181
	Triiodothyronine (T3)	1.2	nmol/L	1.3-3.1
	Free thyroxine (FT4)	9.33	nmol/L	12-22
	Free triiodothyronine (FT3)	2.38	nmol/L	3.1-6.8

Diagnosis: Erysipelas of the lower limbs, sepsis with septic shock, and multiple organ dysfunction syndrome (including acute heart failure, acute kidney injury, and acute liver injury) Rheumatoid arthritis.

### 2.2 Treatment process

Initial stage (D1): Cefuroxime 1.5g was empirically administered intravenously (ivgtt) every 8 hours. However, the patient’s condition rapidly worsened, exhibiting increased agitation, tachypnea, low skin temperature in the extremities, and progressively decreasing blood pressure. Given the diagnosis of septic shock (with a SOFA score of 12), the antibiotic regimen was escalated to meropenem 1g ivgtt q8h in conjunction with moxifloxacin 0.4g ivgtt qd.

ICU Phase (D1–D10): Upon admission to the Intensive Care Unit, pathogen samples (blood and blister fluid) were obtained. Given the strong likelihood of skin infection accompanied by Gram-positive cocci infection, empirical therapy with meropenem 1g ivgtt q8h in combination with teicoplanin 0.4g ivgtt q8h was initiated for anti-infective treatment. Furthermore, the patient exhibited septic shock concurrent with acute heart failure. Picco hemodynamic monitoring revealed a state of high-output, low-resistance, and hypovolemic shock. Management involved restrictive fluid resuscitation and the administration of vasoactive medications (norepinephrine) to sustain blood pressure. For the concurrent multi-organ dysfunction, interventions included non-invasive ventilation, Continuous Renal Replacement Therapy (CRRT), and liver protective measures. Additionally, wound care was administered to the affected areas on the lower limbs (iodophor wet dressing, followed by external application of arnebia oil, augmented by red light therapy). On December 4, 2024, based on the bacterial culture and drug sensitivity test results of the blister fluid, the antibiotic regimen was adjusted to piperacillin/tazobactam 4.5g ivgtt q8h.

**Figure 3:**
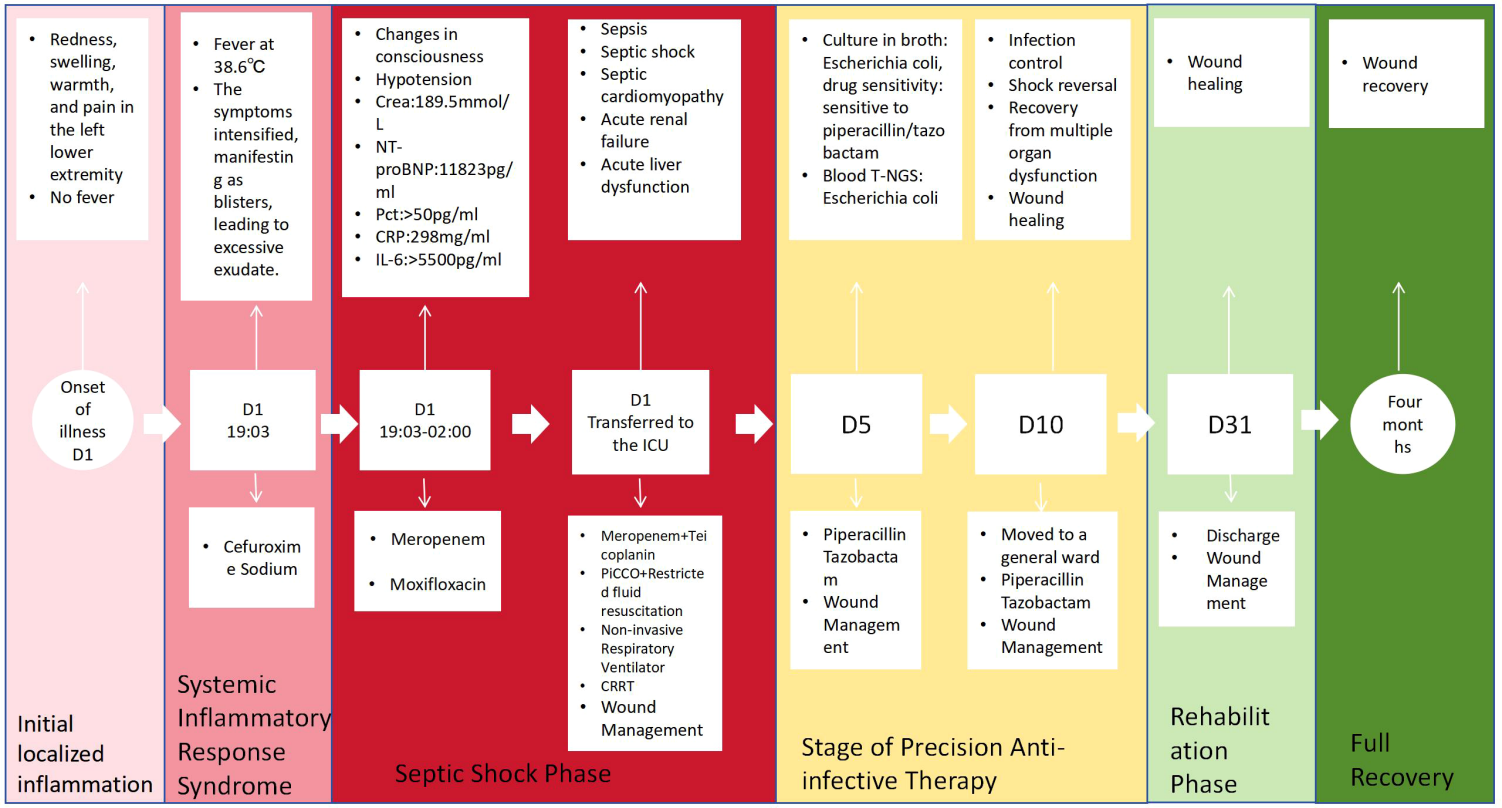
Timeline of clinical course.

Transitional Phase (D10–D31): Upon achieving infection control, shock correction, improvement in multiple organ injuries, and wound healing, the patient was transferred to the General Medicine Department for further treatment. The patient was discharged on December 31, 2024 and subsequently underwent multidisciplinary wound care in the outpatient setting. After a four-month follow-up period, the wound fully healed. A timeline depicting the clinical course is illustrated in **Figure 3**.

## 3 Discussion

Erysipelas and cellulitis constitute acute bacterial infections impacting the skin and subcutaneous tissues. Their hallmark presentations encompass rapidly advancing diffuse edema, erythema, and elevated skin temperature, frequently accompanied by lymphangitis and localized lymphadenopathy. Systemic inflammatory responses encompass fever, tachycardia, altered states of consciousness, hypotension, and leukocytosis. The occurrence of vesicular or bullous skin lesions can exceed 30%. The fundamental pathological distinction between the two conditions resides in the depth of infection: erysipelas predominantly affects the superficial dermis and superficial lymphatic network, whereas cellulitis extends into the deep dermis and subcutaneous fat layer (4). Erysipelas is primarily attributed to beta-hemolytic streptococci (such as group A streptococci), whereas cellulitis is more frequently caused by *Staphylococcus aureus (* 4). However, the pathogen spectrum in immunocompromised hosts demonstrates notable heterogeneity. A study by Eriksson et al. revealed that 21% of erysipelas patients harbored Gram-negative bacteria, with *Escherichia coli* isolated in three cases (5). Notably, *Pseudomonas aeruginosa* can elicit a bullous erysipelas-like vasculitis, further compounding the diagnostic challenge (3). Such infections are particularly prevalent among patients with liver cirrhosis, those who have undergone solid organ transplantation, and individuals with other severe immune deficiencies (6–9), with its incidence rate on the rise. However, it has not been reported in patients with rheumatoid arthritis. Among patients who have received organ transplants, most of those with this type of erysipelas are under the age of 60. Due to host-related factors, their clinical manifestations can vary from severe to mild, typically presenting with fever and skin lesions. The disease generally progresses rapidly and is associated with a poor prognosis. Notably, all three liver transplant patients with spontaneous cellulitis caused by Escherichia coli, as reported by S Janny (9), succumbed to the infection (with an average survival time of 24 hours). Compared to previous cases of E. coli infection, the patient in this case shared a similar immune deficiency status. Nevertheless, this patient had been on immunomodulatory drugs for an extended period, leading to skin thinning due to prolonged corticosteroid use. This resulted in atypical skin manifestations, a more challenging recovery process, and severe complications. However, the patient achieved a complete recovery through aggressive treatment.

The patient in this case is a 61-year-old female with a long-standing history of rheumatoid arthritis, who has been undergoing combined immunosuppressive therapy comprising methylprednisolone, leflunomide, and hydroxychloroquine. She falls into a typical high-risk category. Her clinical course serves as a stark warning: within just 24 hours of onset, localized redness, swelling, heat, and pain in the left lower limb rapidly escalated into widespread blisters/blood blisters (2–3 cm in diameter), accompanied by subcutaneous hemorrhage and pale yellow exudate. This was swiftly followed by septic shock and multiple organ dysfunction, including acute cardiac, hepatic, and renal injuries. This rapid and severe progression underscores the atypical presentation and critical illness propensity of infections in immunosuppressed hosts. Such patients, due to suppressed T-cell function and impaired neutrophil chemotaxis, exhibit not only considerable morphological variations in skin lesions (e.g., necrotic bullae) but are also at an elevated risk of secondary bloodstream infections (with *Escherichia coli* detected in blood t-NGS in this particular case) and multiple organ dysfunction syndrome (MODS) (7).

Traditional culture methods may yield negative results due to low bacterial load, and they also necessitate a longer duration for pathogen identification. After ruling out potential contamination, the pathogen detection rate of NGS is roughly four times higher than that of traditional culture, with positive results obtainable within 48 hours (10, 11). In this instance, *Escherichia coli* infection was concurrently confirmed through blister fluid culture and Next-Generation Sequencing (NGS) technology, underscoring the unique value of NGS in immunosuppressed individuals. NGS does not depend on traditional culture methods; instead, it directly detects all microbial nucleic acids in samples, markedly enhancing detection sensitivity for difficult-to-culture, slow-growing, anaerobic bacteria, pathogens unidentifiable by traditional culture, and those from patients who have undergone antibiotic treatment (10, 12, 13). Consequently, NGS should be promptly adopted as a supplementary diagnostic tool alongside blood and tissue aspirate cultures in severe or immunosuppressed patients.

Furthermore, tiered management of anti-infective treatment necessitates stratified decision-making based on the host’s immune status. For immunocompetent patients, the primary pathogens are β-hemolytic streptococcus or *Staphylococcus aureus*. Penicillin is the preferred treatment for erysipelas, while for cellulitis, antibiotics that cover *Staphylococcus aureus*, such as cephalosporins or clindamycin, are necessary. This strategy achieves a clinical cure rate exceeding 90% by targeting Gram-positive cocci. However, patients with severe conditions or immunosuppression, such as organ transplant recipients or long-term immunosuppressant users, exhibit significant heterogeneity in their pathogen spectrum. These patients require an initial broad-spectrum antibiotic regimen, encompassing coverage for Gram-positive bacteria and drug-resistant Gram-positive bacteria (e.g., vancomycin, linezolid, daptomycin for methicillin-resistant *Staphylococcus aureus*), as well as Gram-negative bacteria and Pseudomonas (using anti-pseudomonas β-lactams such as piperacillin-tazobactam or meropenem). In this particular case, after initial treatment with cefuroxime failed, the regimen was upgraded to meropenem (covering Enterobacteriaceae) in combination with teicoplanin (covering Gram-positive bacteria). Following diagnosis, the treatment was de-escalated to piperacillin-tazobactam. This approach successfully halted the progression of the infection, reinforcing the core principle that immunosuppressed patients must be treated with antibiotics that cover Gram-negative bacteria. Relevant case reports highlight the necessity of early broad-spectrum coverage in organ transplant patients with necrotizing fasciitis or cellulitis caused by Gram-negative bacterial infections, with a mortality rate of 67% when Gram-negative bacteria are not covered (6, 7).Simultaneously, the tiered management approach can markedly enhance patient prognosis. De-escalation therapy ought to be initiated within 24 hours of obtaining pathogen evidence (NGS, bacterial culture). In recent years, the prevalence of drug-resistant *Escherichia coli* has been on the rise. *Escherichia coli* typically demonstrates the following four resistance mechanisms: (1) alterations in membrane permeability; (2) production of enzymes that inactivate the chemical structures of antibacterial drugs; (3) generation of targets that modify the action of antibacterial drugs; (4) upregulation of the expression of active efflux transporters for drugs (14). If no drug-resistance genes or high-virulence genes were detected in this patient, appropriate antibiotic treatment administered throughout the entire course can ensure therapeutic efficacy while minimizing the risk of drug resistance.

Comprehensive wound management also plays a crucial role in consolidating therapeutic outcomes. In this case, local iodine wet compresses and the topical application of purple grass oil for anti-inflammatory effects aided in controlling the *Escherichia coli* infection. When combined with red light therapy, it facilitated angiogenesis and wound healing. The patient had an extremely large overall wound area; wound dried up within one week, and the wound on the left calf healed within one month. However, the wound on the dorsum of the left foot presented healing difficulties. It was considered that, on the one hand, the patient’s prolonged use of glucocorticoids had led to skin atrophy, and on the other hand, the blood supply to the dorsum of the foot was poorer compared to that of the calf. Consequently, wound care was extended to four months.

Patients with immunosuppression exhibiting erysipelas/cellulitis display the following characteristics: a notable shift in the pathogen spectrum, with a marked increase in the proportion of G^−^ bacteria, including Escherichia coli; atypical bullous skin lesions can serve as an early indicator of severe infection, with a rapid clinical course progressing from localized infection to septic shock within a very short timeframe. Consequently, we propose a management pathway for skin and soft tissue infections in immunosuppressed patients: immediate blood and tissue fluid cultures, along with NGS testing for suspected cases; empirical initiation of a broad-spectrum anti-G^−^/G^+^ regimen; antibiotic de-escalation therapy within 24 hours of obtaining pathogen evidence; and comprehensive long-term wound management to consolidate therapeutic outcomes and prevent recurrence. The successful application of this pathway in a case study, where the patient was discharged from the ICU after 9 days and achieved wound healing within 4 months, underscores its clinical significance. Moving forward, further exploration is warranted into individualized anti-infection strategies guided by rapid molecular diagnostic techniques to enhance the prognosis of this high-risk patient population.

## Data Availability

The original contributions presented in the study are included in the article/supplementary material. Further inquiries can be directed to the corresponding author.
